# Internal limiting membrane peel size and macular hole surgery outcome: a systematic review and individual participant data study of randomized controlled trials

**DOI:** 10.1038/s41433-025-03666-9

**Published:** 2025-02-08

**Authors:** Boon Lin Teh, Yanda Li, Keean Nanji, Mark Phillips, Varun Chaudhary, David H. Steel, ILM Peel Size Study Group, Suthasinee Sinawat, Se Woong Kang, Kunho Bae, Hamid Riazi-Esfahani, Elias Khalili Pour, Alireza Khodabande, Jinfeng Qu, Mingwei Zhao, Yuou Yao, Srinivas Sadda, Aditya Modi, Giridhar Anantharaman

**Affiliations:** 1 Sunderland Eye Infirmary, Sunderland, United Kingdom; 2 Biosciences Institute, Newcastle University, Newcastle upon Tyne, United Kingdom; 3 McMaster University, Department of Surgery, Division of Ophthalmology, Hamilton, ON Canada; 4 McMaster University, Department of Health Research Methods, Hamilton, ON Canada; 5 KKU Eye Center, Department of Ophthalmology, Faculty of Medicine, Khon Kaen University, Khon Kaen, Thailand; 6 Samsung Medical Center, Sungkyunkwan University School of Medicine, Seoul, Korea; 7 Seoul National University Hospital, Seoul, Korea; 8 Retina Service, Farabi Eye Hospital, Tehran University of Medical Sciences, Tehran, Iran; 9 Department of Ophthalmology, Peking University People’s Hospital, Beijing, PR China; 10 Doheny Eye Institute, University of California, Los Angeles, CA USA; 11 Modi Eyecare Group, Gwalior, India; 12 Giridhar Eye Insitute, Cochin, India

**Keywords:** Anatomy, Scientific community

## Abstract

**Background:**

There is no consensus regarding the optimal internal limiting membrane (ILM) peel size during vitrectomy for idiopathic full thickness macular holes (iFTMH).

**Methods:**

A systematic review was performed to identify randomized controlled trials (RCTs) comparing vitrectomy with ILM peeling of differing sizes in adults with iFTMH. Individual participant data was obtained including relevant baseline variables. The effect of different ILM peel sizes, divided into “small” (1-disc diameter [DD] in radius or less) and “large” (>1-DD in radius) were analysed on primary hole closure and postoperative visual acuity (VA) at 6 months. A subgroup analysis analysing for the effect of macular hole size on the same outcomes was also performed. Grading of Recommendations, Assessment, Development, and Evaluations (GRADE) was used to assess the certainty of evidence.

**Results:**

Five RCTs with 370 eyes were included. Primary closure was achieved in 74.7% in small peel group compared to 84.8% in large peel group (*p* = 0.016). Multilevel logistic regression showed that a peel radius of >1-DD probably improved hole closure slightly with odds ratio (OR) of 1.20 (95% CI: 1.11–1.31, *p* < 0.001) and a number needed to treat (NNT) to benefit of 31 (95% CI: 21–53). ILM peel size likely did not affect VA. The mean difference in postoperative VA was a -0.05 logMAR gain (2-3 ETDRS letters) (95% CI: −0.13 to 0.02, *p* = 0.155) in vision with a large ILM peel radius. The GRADE certainty of evidence was moderate for both outcomes. A significantly higher closure rate was found in the large peel group for iFTMH >400 microns with an OR of 1.24 (95% CI: 1.11–1.38, *p* < 0.001) and NNT to benefit of 21 (95% CI: 17–50), but not in holes <400 microns (OR 1.05 (95% CI: 0.93–1.18, *p* = 0.396)).

**Conclusions:**

Performing ILM peel of more than 1-DD in radius likely improves closure rates for iFTMH although the effect size is relatively small. The effect is greater in holes >400 microns. ILM peel size probably has no significant effect on postoperative VA.

## Introduction

Idiopathic full-thickness macular holes (iFTMHs) are defects in the foveal centre involving all neural retinal layers [1]. The incidence was reported to range between 3 and 8 per 100,000 per year with prevalence of 0.1–0.8% [2–5]. iFTMHs reduce central visual acuity and have a significant impact on quality of life (QoL) [6].

Surgical management with pars plana vitrectomy (PPV) is successful in closing macular holes and improving visual acuity [7]. Large prospective cohort studies [8, 9] have shown that there are several non-modifiable factors that influence outcomes following surgery. These include pre-operative visual acuity, hole size and the duration of symptoms. Nonetheless, there are several modifiable factors related to surgical techniques that can impact outcomes; most notably internal limiting membrane (ILM) peeling. Peeling of ILM has been demonstrated to significantly improve macular hole closure with an odds ratio (OR) of approximately 9 compared to no peeling [10].

Despite being only a few microns thick, the ILM plays an important role in retinal rigidity and thus it’s removal increases retinal compliance and aids hole closure [11]. ILM peeling however results in a number of effects on inner retinal structure, and potentially function [12]. Transient swelling of the arcuate retinal nerve-fibre layer (SANFL) can occur secondary to instrument-related trauma and dissociated optic nerve-fibre layer (DONFL) can appear a few months postoperatively which is thought to be due to Muller cell avulsion trauma [12–15].

ILM peel size is strongly associated with many of these postoperative changes including the amount of retinal displacement, and it could be hypothesized that larger peels facilitate greater retinal redistribution and hole closure [16]. Indeed, larger ILM peel sizes have been suggested as a method to facilitating closure in revision cases following initial non-closure [17, 18]. Conversely, larger peels may increase the prevalence of the adverse consequences of ILM peeling and it has been suggested that ILM peel size may be optimally adjusted based on iFTMH size [19]. However, there is no consensus regarding optimal peel size; peel sizes between 0.5 and 3-disc diameters (DD) have been described in the literature [16, 19].

Several randomized controlled trials (RCTs) have evaluated ILM peel size in iFTMHs [20–24]. The results have been conflicting, although subgroup analyses in some trials have suggested greater advantages with extended ILM peeling in large holes or those with various shape configurations [21, 23, 24].

Given the present uncertainty, the purpose of this review was to investigate the effect of ILM peel size on the anatomical and functional outcomes following vitrectomy for iFTMH through a systematic review and individual participant data (IPD) analysis study of published RCTs.

## Methods

A systematic search of relevant published scientific literature was first performed to identify eligible RCTs. The systematic review was performed in accordance with the methodology depicted in Cochrane Handbook for Systemic Reviews of Interventions and Preferred Reporting Items for Systematic Reviews and Meta-Analyses (PRISMA) 2020 statement and the protocol was prospectively registered on PROSPERO database (CRD42022306455).

A comprehensive search strategy was developed using appropriate free-text and Medical Subject Headings (MeSH). Studies were identified by using the following electronic bibliographic databases: Ovid (MEDLINE), Ovid (EMBASE), Cochrane Library, Health management information consortium (HMIC), Web of Science, Scopus and trial registers. Bibliographies of the identified studies and of the review articles in the field were searched and reviewed to identify additional relevant studies. All peer-reviewed studies published in the English language were considered. The search date was 6^th^ April 2024.

Inclusion criteria were primary RCTs which had specifically performed standard PPV with ILM peeling at two or more sizes in adults (≥18 years) with iFTMH in association with any of the following manoeuvres: cataract surgery, ILM staining, postoperative positioning protocol and anaesthesia used (local or general). We only included RCTs where both post-surgery anatomical and visual outcomes were assessed, and where hole size, symptom duration and lens status had been recorded.

Exclusion criteria were RCTs that had included secondary macular holes (in association with trauma, retinal detachment, myopia >6 dioptres or retinal dystrophies), non-full thickness macular holes, silicone oil used as tamponade, the use of ILM flaps, persistent iFTMH undergoing reoperation and other diseases which may have affected vision and confound outcomes.

Relevant studies obtained from the search strategy were independently screened by two reviewers (Y.L. and K.N.). Disagreement was solved by discussion or with arbitration by a third reviewer (D.H.S.) until consensus was reached. Following initial screening, eligible studies underwent second-stage full text article screen performed independently by two reviewers to determine eligibility. Disagreements were again solved by discussion or intervention from third reviewer.

For the eligible RCTs, IPD were requested from the corresponding authors via email. The following details were requested; randomization group, age, sex, minimum linear diameter (MLD) in micrometres (microns), base diameter, height of hole, macular hole index (MHI), macular hole closure index (MHCI), symptom duration, phakic status and best corrected visual acuity (BCVA) preoperatively and at 3, 6 and 12 months postoperatively as available, along with primary hole closure status. Measurement of MLD, MHI and MHCI were obtained as illustrated in Supplementary Fig. 1.

Reminder emails were sent if no response were received after two months. Only studies for which IPD could be obtained were included. The type of gas used and postoperative positioning instructions given were obtained from the study protocols. All IPD data from each study were pooled into a single dataset and recoded using a standard coding sheet.

We investigated the effect of different ILM peel sizes divided up into “small” (1-DD in radius or less) and “large” (>1-DD in radius) on two critical outcome measures namely primary macular hole closure (anatomical neurosensory retinal defect (i.e. type 1 closure) using optical coherence tomography to assess end point) and postoperative BCVA at 6 months in all participants and those with primary closure. The size dichotomization was based on 1-DD being a commonly referred to demarcation for small peels [12, 19], and it also being close to the median size of the peel radii used in the studies. Where postoperative BCVA data was not available at 6 months, 6 ± 3 months was used. Important outcome measures considered included quality of life measures, presence of metamorphopsia post surgery and ocular adverse effects if data was collected as part of the original RCT. All BCVA data were converted into logarithm of the minimum angle of resolution (logMAR) units for analysis. Hole sizes were reported as MLD as defined by the International Vitreomacular Traction Study (IVTS) Group classification. We checked for missing, invalid, out-of-range or inconsistent items as well as discrepancies with any trial publication. Any uncertainties were queried with the corresponding authors.

Version 2 of the Cochrane risk-of-bias tool for randomized trials (RoB 2) was adopted for risk of bias assessment. Subsequently, GRADE (grading of recommendations, assessment, development and evaluations) was used to assess the quality and certainty of evidence at the outcome level. Both assessments were performed independently by two reviewers and consensus reached for any disagreements by discussion.

### Statistical analysis

A multivariable logistic regression model was used to assess factors associated with anatomical closure. Confounding factors, based on previous literature [8, 25, 26] were included in the model including age, choice of tamponade, preoperative BCVA, symptom duration, days of postoperative face down positioning, MLD, and pre- and postoperative phakic status. Study centre was also included in the model. Results are expressed as OR with 95% confidence intervals (CI). Similarly, a linear regression model with the same adjusted confounding factors was used to study the effect of ILM peel size on postoperative BCVA in all participants and those who achieved primary iFTMH closure. Multicollinearity was considered if the variance inflation factor (VIF) for any variable was greater than 1.5. If interactions were suspected based on prior evidence, interaction terms were evaluated within the models. No data transformations were performed, all data were analysed as reported within the respective studies. Random effects variance was assessed and interpreted for each regression model. Random effects variance corresponds to the extent of variance or heterogeneity observed between the studies included. This value is important in understanding the extent that the results could be attributable to variance in study conduct, design, execution, or other study-related factors. There are no clearly defined cutoffs to consider heterogeneity as “low” or “high”, but instead these values can be interpreted as the extent that differences in the outcome variable can be attributable to the study variable itself.

We also conducted an unplanned subgroup analysis on the effect of macular hole size on closure and postoperative visual with the two ILM peel sizes based on a macular hole size of 400 microns, as well as size bands of <300, 301–400, 401–500, 501–600 and >600 microns. Analyses were conducted with R software (Version 4.2.2).

## Results

A total of 2740 studies were initially identified. Five RCTs that met our inclusion criteria were included (Supplementary Fig. 2). IPD was requested and successfully obtained from all authors.

Details of the 5 RCTs included in the analysis are displayed in Table 1, along with their baseline characteristics shown in Supplementary Table 1.

**Table 1:** All studies included in individual participant data analysis.

Study (primary author, published date)	Funder	Eyes/patients per Study (N)	Included iFTMH sizes (micron)	Maximum symptom duration (months)	Intraocular tamponade agent	pPH or PhV at baseline (N)	Postoperative positioning instructions	Visual acuity assessment method	ILM peel radii group
Yao et al. [24], China	Beijing Municipal Science and Technology Commission. National Natural Science Foundation of China Grant.	121/121	All sizes	≤36	20% SF_6_	1 pPH/102 PhV	FDP 14 days	Refraction/ETDRS chart	1-DD or 2-DD
Bae et al. [20], South Korea	Departmental funding only	59/59	All sizes	No limits	25% SF_6_ (<400 micron with <3 months symptoms) otherwise 14% C_3_F_8_	6 pPH15 PhV	FDP 5 days	BCVA/ETDRS chart	0.75-DD or 1.5-DD
Khodabande et al. [21], Iran	Departmental funding only	40/39	All sizes	≤ 36	20% SF_6_	10 pPH30 PhV	FDP 3 days	BCVA/logMAR	1-DD or 2-DD
Modi et al. [22], India	Departmental funding only	50/50	All sizes	No limits	20% SF_6_	8 pPHNo PhV	FDP 5 days	BCVA/Snellen converted to logMAR	1-DD or 1.5-DD
Sinawat et al. [23], Thailand	Invitation Research Fund, Faculty of Medicine, Khon Kaen University	100/100	>400 microns only	No limits	20% SF_6_	19 pPH25 PhV	FDP 1-2 weeks	BCVA/logMAR	1-DD or 2-DD

Key descriptive characteristics of the included randomized controlled trial studies included in the individualized participant data analysis.

*BCVA* best-corrected visual acuity, *C*_*3*_*F*_*8*_ octafluoropropane, *DD* disc diameter, *ETDRS* Early Treatment Diabetic Retinopathy Study, *FDP* face-down positioning, *iFTMH* idiopathic full-thickness macular hole, *ILM* internal limiting membrane, *logMAR* logarithm of the minimum angle of resolution, *PhV* combined phacovitrectomy, *pPH* pseudophakic, *SF*_*6*_ sulfur hexafluoride, *microns* micrometres.

Overall, 370 eyes of 369 patients were included in this study. The median age was 64 years (interquartile range [IQR], 8), and duration of symptoms at the time of surgery was 4 months (IQR, 9). Four trials included iFTMH of all sizes and one recruited only those larger than 400-micron MLD. The median MLD was 492.5 micron (IQR, 308.5) and preoperative BCVA was 1.0 logMAR (Snellen equivalent: 6/60). There were 241 eyes greater than 400 microns in MLD. The baseline characteristics of the two intervention groups were similar (Supplementary Table 2). 186 eyes were randomized into the small ILM peel group and 184 in the large ILM peel group.

**Fig. 1:**
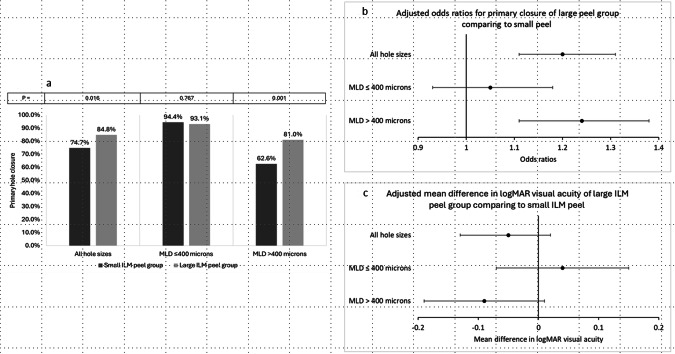
Primary and adjusted anatomic and visual outcomes. **a** Bar chart comparing primary hole closure rates between small and large peel group in all hole sizes, hole sizes ≤400 microns and >400 microns. **b** Adjusted odds ratio (with corresponding 95% confidence interval) for primary hole closure of large peel group comparing to small peel group in all hole sizes, hole sizes ≤400 microns and >400 microns. **c** Adjusted mean difference (with corresponding 95% confidence interval) in postoperative logMAR visual acuity of large peel group comparing to small peel group in all hole sizes, hole sizes ≤400 microns and >400 microns.

Overall primary hole closure was achieved in 79.7%. Primary iFTMH closure was achieved in 74.7% of eyes in the small ILM peel group compared to 84.8% of eyes in the large ILM peel group (*p* = 0.016) (Fig. 1a).

### Adjusted outcomes

#### Anatomical closure

Data on anatomical closure was available in all 370 eyes. Baseline data was complete for included variables except MHI (243 eyes) and MHCI (274 eyes). MLD was highly correlated with MHCI and MHI (Supplementary Figs. 3 and 4) and hence regressions were conducted including only one of these values at a time.

Multivariable logistic regression, including MLD and other variables, showed that a peel radius of greater than 1-DD increased the occurrence of primary hole closure with an OR of 1.20 (95% CI: 1.11–1.31, *p* < 0.001) (Fig. 1b). The number needed to treat (NNT) to benefit was 31 (95% CI: 21–53) i.e. 31 patients treated with a large peel would be needed to observe 1 additional closure as compared to a small peel. The absolute effect per 1000 eyes was 747 closures with a small peel and 780 (95% CI: 767–795) with a large peel meaning there would be 33 additional closures with a large peel per 1000 patients.

The random effects variance between studies was low at σ^2^ = 0.12 suggesting low heterogeneity of effect between participants. MLD (OR 0.94 per 100 microns (95% CI 0.92–0.96, *p* < 0.001)) and symptom duration (OR 0.99 per month (95% CI 0.98–99, *p* < 0.001)) were also significantly associated with closure.

When MHCI was included in the model instead of MLD (*n* = 274), the OR for peel size was similar at 1.17 (95% CI: 1.08–1.27), and with MHI included (*n* = 243), it was 1.31 (95% CI: 1.15–1.50); with *p* < 0.001 for both. In these models MHCI was significantly associated with closure (OR = 1.03; 95% CI: 1.01–1.05; *p* = 0.004), whilst MHI was not (OR = 1.02; 95% CI: 1.00–1.04; *p* = 0.081).

### Visual acuity

The median baseline BCVA was similar between the two groups at 1.0 logMAR (Snellen equivalent: 6/60). Postoperative visual acuity was available for 363 of the 370 eyes in total and the analysis was carried out on these 363 eyes. Adjusted for baseline BCVA and other confounders including lens status pre and postoperatively, there was no significant difference in visual outcome between the two groups with a mean gain of −0.05 logMAR (95% CI: −0.13 to 0.02, *p* = 0.155) (i.e. 2-3 ETDRS letters) with a large peel radius (Fig. 1c). The random effects variance between studies was again low at σ^2^ = 0.08. MLD (OR 0.06 per 100 microns (95% CI 0.03–0.08, *p* < 0.01)), preoperative visual acuity (OR 0.22 per 1.0 logMAR (95% CI 0.11–0.33, *p* < 0.001)) and phakic status (OR 0.11 (95% CI 0.02–0.20, *p* = 0.018)) were also significantly associated with postoperative visual acuity.

The results were similar when the analysis was restricted to those with primary closure with a mean loss of 0.05 logMAR (95% CI: −0.03 to 0.12, *p* = 0.208) with a large peel.

### Subgroup analysis: effect of MLD on primary closure and visual acuity

Using the IVTS classification of 400 microns for large iFTMH, there was a significantly higher unadjusted closure rate in the large ILM peel group for iFTMH >400 microns (62.6% closure in the small peel group, *n* = 115 versus 81% in the large peel group, *n* = 126; *p* = 0.001), but not for holes less than 400 microns (94.4%, *n* = 71 versus 93.1%, *n* = 58; *p* = 0.767) (Fig. 1a).

In the group of holes >400 microns, multivariable logistic regression, including MLD and other variables, showed that a peel radius of greater than 1-DD increased the occurrence of primary hole closure with an OR of 1.24 (95% CI: 1.11–1.38, *p* < 0.001) as compared to a smaller peel (Fig. 1b). The NNT to benefit was 21 (95% CI: 17–50) i.e. 21 patients treated with a large peel would be needed to observe 1 additional closure as compared to a small peel. The mean difference in visual acuity was a gain of −0.09 logMAR (95% CI: −0.19 to 0.01, *p* = 0.074 (i.e. 4-5 ETDRS letters) with a large peel (Fig. 1c).

For eyes with iFTMH less than 400 microns, the OR for primary closure was non-significant at 1.05 (95% CI: 0.93–1.18, *p* = 0.396) (Fig. 1b), with no significant effect on visual acuity with a loss of 0.04 logMAR (95% CI: −0.07 to 0.15, *p* = 0.465) (Fig. 1c).

Further subgroup analysis stratifying MLD into 100 microns increments from 300 to 600 microns is shown in Table 2. It was noted that closure rate for iFTMH of size 501–600 microns was significantly higher in the large ILM peel group. Using receiver operating characteristic (ROC) analysis, the optimal MLD threshold (Youden index) for a large peel was 497 microns (sensitivity: 0.59, specificity: 0.85) (Supplementary Fig. 5).

**Table 2:** Primary iFTMH closure rates at 100 microns MLD increment for small and large ILM peel groups.

MLD (microns)	Overall closure (%)	Small ILM peel (*n* = 186) % primary closure	Large ILM peel (*n* = 184) % primary closure	Unadjusted *p*-value	Odds Ratio (95% CI)
≤300 (*n* = 72)	95.8	97.4 (*n* = 39)	93.9 (*n* = 33)	0.459	1.00 (0.87–1.15)
301–400 (*n* = 57)	91.2	90.6 (*n* = 32)	92 (*n* = 25)	0.856	1.06 (0.87–1.31)
401–500 (*n* = 60)	91.7	86.7 (*n* = 30)	96.7 (*n* = 30)	0.161	1.10 (0.94–1.27)
501–600 (*n* = 72)	77.8	64.3 (*n* = 28)	86.4 (*n* = 44)	0.028	1.31 (1.09–1.58)
>600 (*n* = 109)	57.8	49.1 (*n* = 57)	67.3 (*n* = 52)	0.055	1.18 (0.99–1.42)

*iFTMH* idiopathic full thickness macular hole, *ILM* internal limiting membrane, *MLD* minimum linear diameter.

No studies published data on quality of life. Similarly, only one study published data on metamorphopsia, and none published adverse events and hence we have not presented data on those outcomes.

### Study risk of bias

**Fig. 2:**
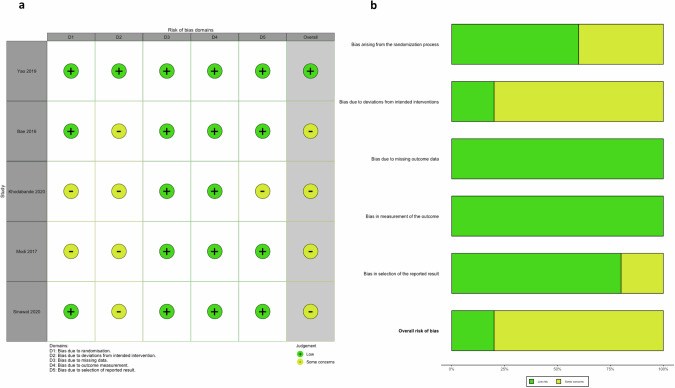
Risk of bias. **a** Risk of bias traffic light plot for macular hole closure rate and postoperative visual acuity. **b** Risk of bias summary figure for macular hole closure rate and postoperative visual acuity.

The RoB 2 tool was used to examine the risk of bias for all included studies for both critical outcomes. Out of the 5 studies, one [24] was judged at low risk of bias overall, and 4 studies had some risk of bias concerns for both macular hole closure and postoperative visual acuity (Fig. 2). There were some concerns regarding how the trials controlled and ensured ILM peel size was performed in accordance with randomisation group. While peel size was mainly gauged based on operating surgeon’s perception during operation, only one study [24] independently verified the ILM peel size following surgery. Two studies [21, 22] also did not describe the method of random allocation.

Using the GRADE approach, which is a systematic approach to rating the certainty of evidence in systematic reviews [27] we graded the overall certainty of evidence for the included studies as moderate for hole closure and visual acuity (Table 3). We downgraded the certainty of evidence by one level for overall risk of bias concerns in 4 of the 5 studies.

**Table 3:** GRADE summary of findings: efficacy of large ILM peel size versus versus small ILM peel size for idiopathic FTMH.

Population: Adults with idiopathic FTMH. Setting: International eye centres in 5 countries globally. Intervention: Large ILM peel size combined with vitrectomy and gas tamponade. Comparison: Small ILM peel size combined with vitrectomy and gas tamponade.					
Outcomes	Comparative outcome measures (95% CI)		Relative effect (95% CI)	Number of participants (studies)	Certainty of the evidence (GRADE)
	Assumed outcome with comparator	Corresponding outcome with intervention			
Rate of successful primary macular hole closure	747 per 1000	780 per 1000 (95% CI 767–795) NNT 31 (21–53)	OR 1.20 (1.11–1.31, *P* < 0.001)	370 (5)	⨁⨁⨁◯^A^ Moderate
Visual acuity at 6 months after surgery or nearest timepoint (logMAR)	After surgery, the visual acuity in the small ILM peel group ranged from −0.04 logMAR (best vision) to 1.78 logMAR (worst vision)	After surgery, the visual acuity in the large ILM peel group, adjusted for baseline vision, improved by −0.05 logMAR (95% CI: −0.13 to 0.02, *P* = 0.155)	−0.05 logMAR (95% CI: −0.13 to 0.02, *p* = 0.155).	363 (5)	⨁⨁⨁◯^B^ Moderate

GRADE Certainty of evidence – High‐certainty: Further research is very unlikely to change our confidence in the estimate of effect. Moderate‐certainty: Further research is likely to have an important impact on our confidence in the estimate of effect and may change the estimate. Low‐certainty: Further research is very likely to have an important impact on our confidence in the estimate of effect and is likely to change the estimate. Very low‐certainty: Any estimate of effect is very uncertain.

Reasons for downgrading certainty of evidence: ^A^Rated down (−1 levels) for concerns regarding risk of bias in 4 out of the 5 included studies. ^B^Rated down (−1 levels) for concerns regarding risk of bias in 4 out of 5 studies.

*CI* confidence interval, *ILM* internal limiting membrane, *FTMH* full-thickness macular hole, *GRADE* grading system for evidence and recommendations, *OR* odds ratio.

## Discussion

This IPD meta-analysis of 5 RCTs, which included 370 eyes showed that an ILM peel of 1-DD or more in radius during iFTMH surgery probably results in higher rates of hole closure than a smaller peel. The effect size was small with a NTT to benefit of 31. There was a corresponding small gain in vision with a large peel radius of −0.05 logMAR (95% CI: −0.13 to 0.02, *p* = 0.155). The effect was independent of postoperative posturing, gas type, age or duration of iFTMH. A subgroup analysis showed that the effect ILM peel radii on hole closure and postoperative visual acuity was greater in larger holes and became more important above 400 microns in MLD in terms of closure, where the NNT to benefit reduced to 21.

The results of individual RCTs assessing ILM peeling size have been unclear with differing results (Supplementary Table 3). The size of holes included which is a key determinant of closure has been quite variable. Sinawat et al. [23] exclusively included holes greater than 400 microns with a median MLD of 623 microns, whilst in the trial by Bae et al. [20], the median MLD of the holes was approximately 288 microns only. Similarly, the chronicity of the holes has been variable, and is a known factor in closure and visual outcome [25]. Sinawat et al. [23] included all durations, with a median of 11 months, whilst duration of iFTMH of other 4 RCTs were much shorter with a median of 6 months or less [20–22, 24]. Relevantly, none of the RCTS were powered with adequate sample size to assess a clinically relevant benefit on hole closure rates or exclude a clinically significant effect on visual acuity outcomes. Aggregate meta-analyses using summary-level data are prone to confounding from trial population heterogeneity whilst an individual participant data approach can standardize outcomes and covariates across trials, borrowing statistical strength from smaller trials, improving the robustness of analyses and allowing more reliable comparisons and explorations of treatment modifiers such as hole size [28–30]. In particular the included RCTs included a range of macular hole sizes which would make aggregate metanalysis unreliable.

By analysing a combined individual participant dataset, we have been able to make clear estimates of the effect of ILM peeling size on closure and visual acuity, considering the effects of important variables including hole size and symptom duration. Overall, our IPD analysis shows that a large ILM peel probably improved hole closure, but our subgroup analysis suggested the effect was greater and more clinically relevant for closure above 400 microns. It could be considered that 400 microns and above would be a clinically relevant size point at which surgeons should enlarge peel radii to above 1-DD, based on the 10% difference in closure rates observed. A 10% difference in closure rates has been commonly used in trials as being a ‘minimal importance difference’. None of the studies used a peel radius of greater than 2-DD in size which is the largest peel radius that can be recommended. Interestingly, the size of hole where the effect of a larger peeling radius was optimally predictive for closure was at approximately 500 microns, which is the same threshold which others have found marks a clinically evident reduction in iFTMH closure rates with conventional surgery. We also found the same effect with overall closure rates declining from above 90% below 500 microns, to 78% above 500 microns [8, 31]. As hole size increased beyond 500 microns, a large peel radius continued to improve closure rates despite a gradually decreasing overall closure rate. The threshold at which surgeons choose to perform other ancillary procedures to improve closure such as ILM flaps is debated, but 500 microns has also been proposed [32–34].

Multiple indices have been proposed to prognosticate macular holes. However, there is limited supporting evidence to suggest that any of these calculated measurements have a higher level of accuracy and advantage in predicting outcomes as they are highly co-linear [26]. We found MHCI, but not MHI was associated with closure. In macular holes with an MHCI of less than 0.5, a large peel was associated with improved closure. However, plotting MHCI versus MLD showed that there were very few holes of less than 400 microns where the MHCI was less than 0.5 (Supplementary Fig. 3). MLD is a widely used measure and adding MHCI would contribute only marginally to decision making.

We did not find any definite statistically significant effect of peel size on visual outcomes. However overall, with all cases included there was a mean gain of −0.05 logMAR with a large peel likely related to the higher closure rates achieved supported by the finding that this increased to a gain of −0.09 logMAR when restricted to holes above 400 microns. It should also be observed that there was a non-significant loss of 0.05 logMAR in the large peel group in those with primary closure, and we were unable to exclude a detrimental effect of large peel size with the confidence limits spanning from −0.03 to 0.12 logMAR. Relevantly we did not have data on other measures of foveal function which may be affected by ILM peel size including microperimetry, visual fields and electrodiagnostic tests.

Metamorphopsia and QoL were also not included, as only one study measured metamorphopsia [20], and none included QoL both important for guideline development and weakening the clinical relevance of our findings. Bae et al. [20] found that larger peels were associated with a greater improvement in metamorphopsia following surgery. This was attributed to lesser asymmetry of foveal elongation in the larger ILM peel group. Further investigation of these aspects would be needed to provide definitive evidence.

We accept that our study has some limitations other than those already discussed. Only one study [24] included an independent verification of ILM peel size, and the other studies relied on surgeon judgement which lowers the certainty of the evidence. None of the included RCTs excluded patients with epiretinal membrane and only one [23] recorded its presence. Therefore, we were unable to systematically control for its effects and it is possible it could have confounded the analysis. One study [22] also did not describe blinding of outcome assessors which could introduce some bias. We included ‘study’ as a level in our modelling to account for heterogeneity between studies but did not find any significant effect. Although the geographical spread of countries included was large, there were none from the USA or Europe. It is known that ethnicity can affect macular hole outcomes, and this could affect the generalisability of our results. We excluded trials on persistent and secondary holes and in those where oil was used, and our results cannot be used to guide decision making in these cases. Lens management differed between studies and could have confounded our results, but preoperative and postoperative lens status were included as variables. We performed an unplanned analysis on the modulating effect of hole size on closure with different ILM peel radii. Being not pre-planned the results of this analysis need to be interpreted with caution.

In conclusion this IPD meta-analysis of 370 eyes with iFTMH found that performing ILM peel of more than 1-DD in radius probably improves closure rates for iFTMH and visual outcomes but the effect is small with a NNT of 31 and a mean gain of −0.05 logMAR only. The GRADE certainty of evidence was moderate meaning that future evidence may alter the effect estimates. A subgroup analysis suggests that the effect was more pronounced in holes above 400 microns or more in MLD, reaching its greatest effect in holes of approximately 500 microns.

## Summary

### What was known before

- Vitrectomy with ILM peeling is successful in closing macular hole and improving visual acuity. However, there is no consensus regarding the optimal peel size to be performed.

### What this study adds

- An ILM peel of more than 1-disc diameter in radius probably improves closure rates for full thickness macular hole as compared to smaller peel radii.
- The effect is small with a NNT to benefit of 31 for closure. The effect of visual acuity was also small with a mean gain of −0.05 logMAR.
- The effect was greater for holes greater than 400 microns in MLD where the NNT reduced to 21.

## Supplementary information

Supplementary Table 1

Supplementary table 2

Supplementary table 3

Supplementary Figure 1

Supplementary Figure 2

Supplementary Figure 3

Supplementary Figure 4

Supplementary Figure 5

Figure Legends

## Data Availability

The datasets generated during and/or analysed during the current study are available from the corresponding author on reasonable request.
