# Supplementary material for: Internal limiting membrane peel size and macular hole surgery outcome: a systematic review and individual participant data study of randomized controlled trials
Source: Eye (Lond). 2025 Feb 8;39(7):1406–13. doi: 10.1038/s41433-025-03666-9 (PMC12044072; doi:10.1038/s41433-025-03666-9)
Supplement: Supplementary file 1 — Supplementary Table 1 [file 41433_2025_3666_MOESM1_ESM.docx]

**Supplementary Table 1: Baseline characteristics of included studies**

| **Baseline characteristics** | | | | | | |
| --- | --- | --- | --- | --- | --- | --- |
| **Study** | **No. of Eyes Included** | **Age, years  (Median, Range)** | **MLD, microns  (Median, Range)** | **Duration, months (Median, Range)** | **Pre-operative BCVA, logMAR (Median, Range)** | **Post-operative BCVA* at 6 ± 3 months, logMAR (Median, Range)** |
| **Yao et al, 2019** | 121 | 65 (45-78) | 468 (127-1050) | 2 (0.25-36) | 0.78 (0.22-1.40) | 0.41 (0.04 – 1.40) |
| **Bae et al, 2016** | 59 | 64 (39-78) | 288 (71-610) | 1 (0.2-12) | 0.70 (0.28-1.26) | 0.26 (0.00 – 1.00) |
| **Khodabande et al, 2020** | 40 | 64 (53-89) | 525.5 (130-790) | 6 (1-36) | 1.00 (0.30-2.00) | 0.40 (0.05 – 1.52) |
| **Modi et al, 2016** | 50 | 66 (46-77) | 359 (43-822) | 2.5 (0.37-12.2) | 0.90 (0.40-1.50) | 0.60 (0.20 – 1.40) |
| **Sinawat et al, 2020** | 100 | 62 (40-90) | 623.5 (404-1045) | 11 (2-72) | 1.03 (0.60-2.00) | 0.91 (0.00 – 1.86) |
| **Combined dataset** | 370 | 64 (39-90) | 492.5 (43 - 1050) | 4 (0.2-72) | 1.00 (0.22-2.00) | 0.50 (0.00 – 1.86) |

BCVA = best-corrected visual acuity; logMAR = logarithm of the minimum angle of resolution; MLD = minimum linear diameter

*Missing data on 7 post-operative BCVA
