# Supplementary material for: Internal limiting membrane peel size and macular hole surgery outcome: a systematic review and individual participant data study of randomized controlled trials
Source: Eye (Lond). 2025 Feb 8;39(7):1406–13. doi: 10.1038/s41433-025-03666-9 (PMC12044072; doi:10.1038/s41433-025-03666-9)
Supplement: Supplementary file 2 — Supplementary table 2 [file 41433_2025_3666_MOESM2_ESM.docx]

**Supplementary table 2: Characteristics of all patients with iFTMH in each treatment arm**

|  | **Small (1-disc diameter or less in radius) ILM peel, n=186** | **Large (>1 disc diameter in radius) ILM peel, n=184** |
| --- | --- | --- |
| Age, years. (Median, IQR) | 65 (10) | 64 (9) |
| Female sex. (n, %) | 132 (71.0) | 135 (73.4) |
| Symptom duration, months. (Median, IQR) | 3 (8.8) | 4 (9) |
| MLD, microns. (Median, IQR) | 471 (323) | 513 (267) |
| MHCI (Median, IQR) | 0.56 (0.29) | 0.56 (0.26) |
| MHI (Median, IQR) | 0.48 (0.22) | 0.46 (0.24) |
| Phakic pre-operative. (n, %) | 165 (88.7) | 162 (88.0) |
| Phakic post-operative. (n, %) | 70 (37.6) | 74 (40.2) |
| Baseline BCVA, logMAR (Median, IQR) | 1.00 (0.60) | 0.95 (0.52) |
| Primary iFTMH closure. (n, %) | 139 (74.7) | 156 (84.8) |
| Post-operative BCVA, logMAR. (Median, IQR) | 0.50 (0.64) | 0.52 (0.52) |

BCVA = best-corrected visual acuity; iFTMH = idiopathic full thickness macular hole; ILM = internal limiting membrane; IQR = interquartile range; logMAR = logarithm of the minimum angle of resolution; MHCI = macular hole closure index; MHI = macular hole index; MLD = minimum linear diameter
