# Supplementary material for: Internal limiting membrane peel size and macular hole surgery outcome: a systematic review and individual participant data study of randomized controlled trials
Source: Eye (Lond). 2025 Feb 8;39(7):1406–13. doi: 10.1038/s41433-025-03666-9 (PMC12044072; doi:10.1038/s41433-025-03666-9)
Supplement: Supplementary file 3 — Supplementary table 3 [file 41433_2025_3666_MOESM3_ESM.docx]

**Supplementary table 3. Summary table of primary hole closure and visual outcome in included trials.**

| **Study** | **MLD, microns  (Median, Range)** | **Small ILM peel group number of eyes** | **Large ILM peel group number of eyes** | **Small ILM peel group closure rate** | | | **Large ILM peel group closure rate** | | | **Mean difference between large and small ILM peel group in post-operative BCVA* at 6 ± 3 months, logMAR** | | |
| --- | --- | --- | --- | --- | --- | --- | --- | --- | --- | --- | --- | --- |
|  |  |  |  | **Overall** | **MLD** **≤400 microns** | **MLD > 400 microns** | **Overall** | **MLD** **≤400 microns** | **MLD >** **400 microns** | **Overall** | **MLD** **≤400 microns** | **MLD >** **400 microns** |
| **Yao et al, 2019** | 468 (127-1050) | 63 | 58 | 82.5% | 91.7% | 76.9% | 91.4% | 95.2% | 89.2% | +0.02 | +0.06 | -0.01 |
| **Bae et al, 2016** | 288 (71-610) | 30 | 29 | 100% | 100% | 100% | 100% | 100% | 100% | +0.03 | +0.06 | -0.04 |
| **Khodabande et al, 2020** | 525.5 (130-790) | 19 | 21 | 52.6% | 75.0% | 36.4% | 76.2% | 100% | 73.7% | -0.20 | -0.30 | -0.28 |
| **Modi et al, 2016** | 359 (43-822) | 25 | 25 | 80.0% | 86.7% | 70.0% | 64.0% | 73.3% | 50.0% | +0.24 | +0.19 | +0.28 |
| **Sinawat et al, 2020** | 623.5 (404-1045) | 49 | 51 | 51.0% | N/A | 51.0% | 76.5% | N/A | 76.5% | -0.10 | N/A | -0.10 |

BCVA = best-corrected visual acuity; logMAR = logarithm of the minimum angle of resolution; MLD = minimum linear diameter

*Missing data on 7 post-operative BCVA
