# Supplementary figures and images for: Internal limiting membrane peel size and macular hole surgery outcome: a systematic review and individual participant data study of randomized controlled trials
Source: Eye (Lond). 2025 Feb 8;39(7):1406–13. doi: 10.1038/s41433-025-03666-9 (PMC12044072; doi:10.1038/s41433-025-03666-9)

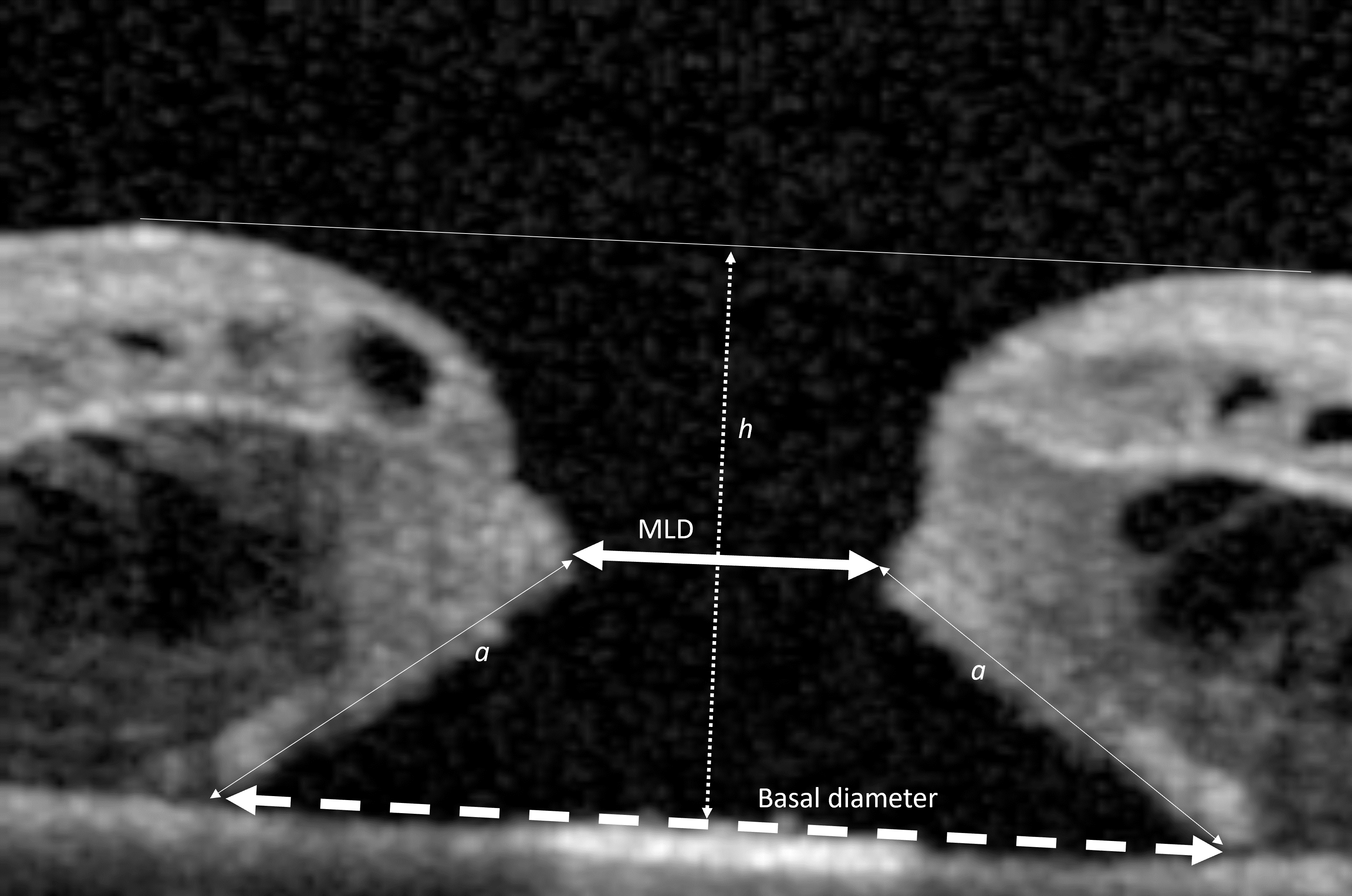

Supplement: Supplementary file 4 — Supplementary Figure 1 [file 41433_2025_3666_MOESM4_ESM.png]

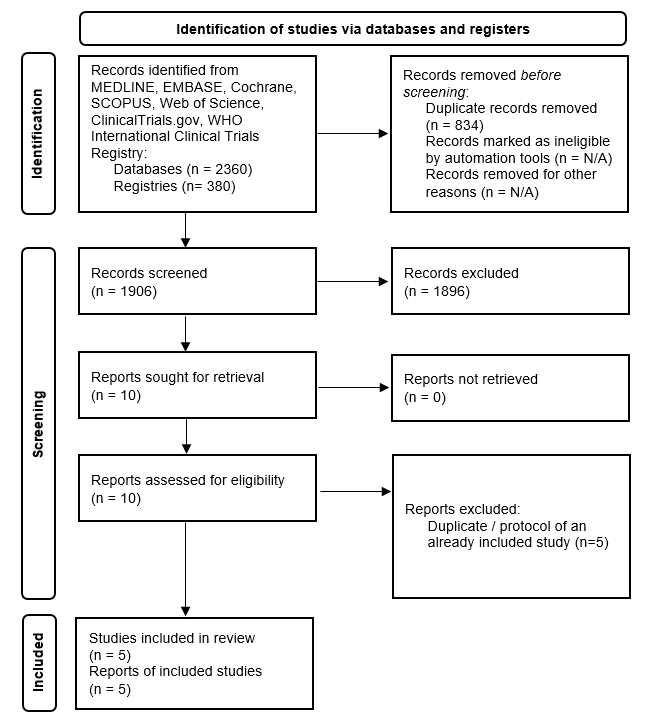

Supplement: Supplementary file 5 — Supplementary Figure 2 [file 41433_2025_3666_MOESM5_ESM.tif]

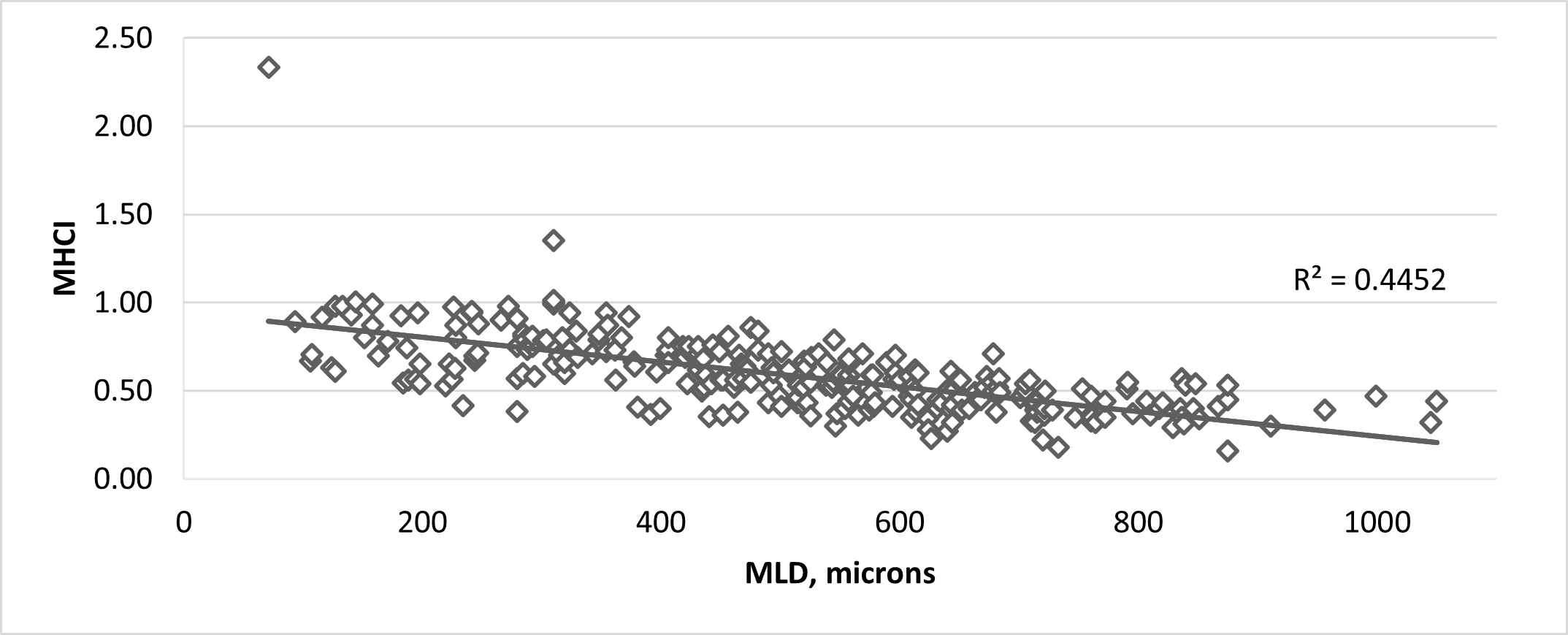

Supplement: Supplementary file 6 — Supplementary Figure 3 [file 41433_2025_3666_MOESM6_ESM.png]

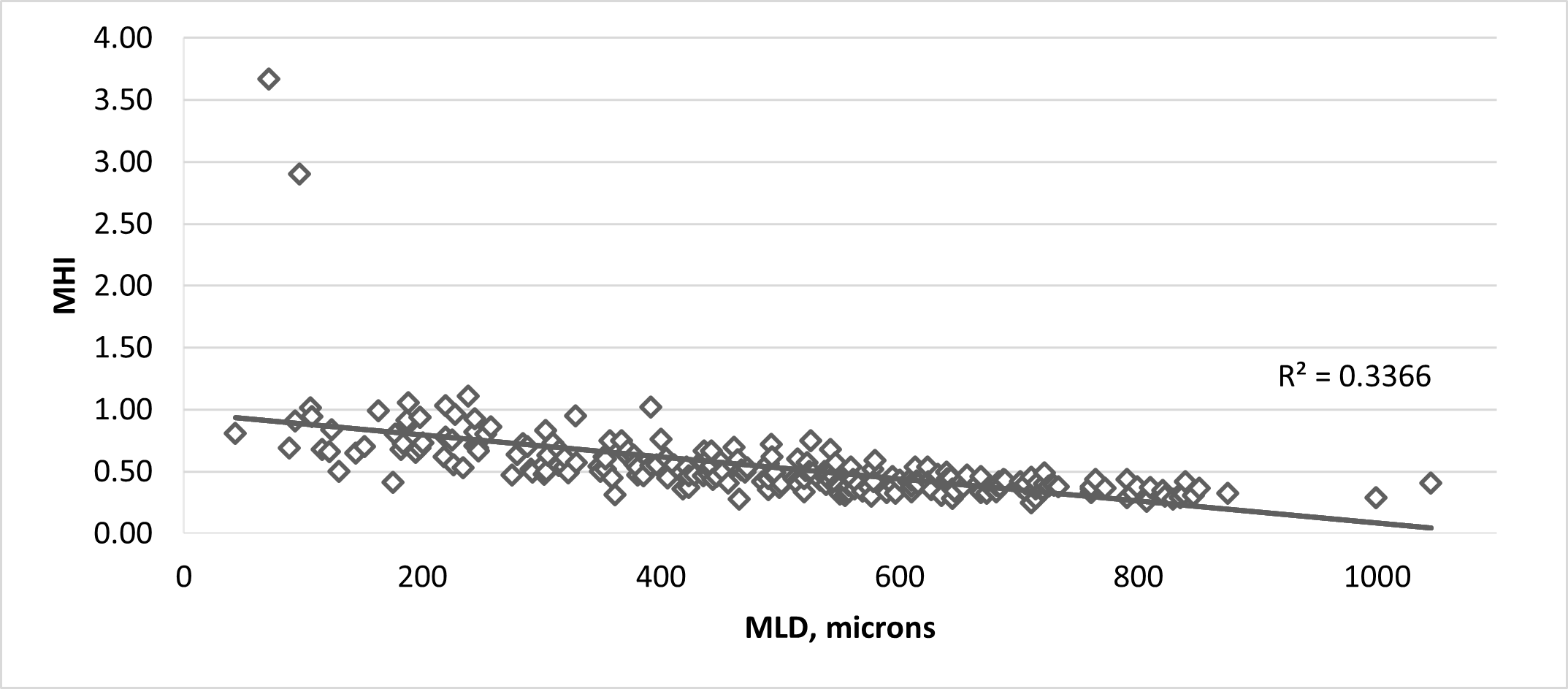

Supplement: Supplementary file 7 — Supplementary Figure 4 [file 41433_2025_3666_MOESM7_ESM.png]

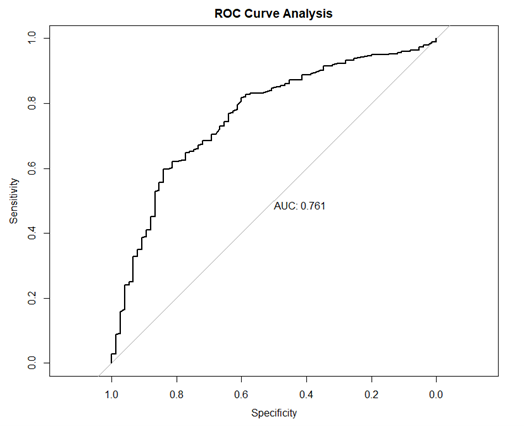

Supplement: Supplementary file 8 — Supplementary Figure 5 [file 41433_2025_3666_MOESM8_ESM.png]
