# Supplementary material for: Internal limiting membrane peel size and macular hole surgery outcome: a systematic review and individual participant data study of randomized controlled trials
Source: Eye (Lond). 2025 Feb 8;39(7):1406–13. doi: 10.1038/s41433-025-03666-9 (PMC12044072; doi:10.1038/s41433-025-03666-9)
Supplement: Supplementary file 9 — Figure Legends [file 41433_2025_3666_MOESM9_ESM.docx]

Supplementary Figure 1: Minimum linear diameter (MLD) is the horizontal diameter at the narrowest point. Macular hole index (MHI) is calculated using perpendicular height (*h*) divided by basal diameter. Macular hole closure index (MHCI) is calculated as the sum length of the detached photoreceptor arms (*a*) divided by basal diameter.

Supplementary Figure 2: Preferred Reporting Items for Systematic Reviews and Meta-Analyses (PRISMA)-compliant flowchart that shows the number of studies identified following the search strategy.

Supplementary Figure 3: Relationship between MLD and MHCI.

Supplementary Figure 4: Relationship between MLD and MHI.

Supplementary Figure 5: Receiver operating characteristic (ROC) curve analysis for primary closure by MLD.
